# Influence of the Degradation Medium on Water Uptake, Morphology, and Chemical Structure of Poly(Lactic Acid)-Sisal Bio-Composites

**DOI:** 10.3390/ma13183974

**Published:** 2020-09-08

**Authors:** Cristina Moliner, Elisabetta Finocchio, Elisabetta Arato, Gianguido Ramis, Alberto Lagazzo

**Affiliations:** Department of Civil, Chemical and Environmental Engineering (DICCA), University of Genoa, via all’Opera Pia 15, I-16145 Genova, Italy; elisabetta.arato@unige.it (E.A.); gianguidoramis@unige.it (G.R.); alberto.lagazzo@unige.it (A.L.)

**Keywords:** PLA/sisal bio-composites, water absorption, IR spectroscopy, microstructure, pH

## Abstract

A series of poly(lactic acid) (PLA) and poly(lactic acid)-based bio-composites (sisal PLA) were prepared and studied by spectroscopic and microscopic techniques as such and after immersion at room temperature in different degradation mediums (i.e., distilled and natural sea water and solutions at pH = 2, 6, and 8). In these conditions, some of their macroscopic and microscopic properties were monitored during a period of 30 days. Water absorption increased with the increasing fiber content regardless of the immersion medium. The maximum water absorption was achieved at pH = 8 (~16%), indicating a more severe action of the alkaline mediums on the samples. The diffusivity, *D*, of PLA decreased with the addition of fibers and acidic mediums showed higher *D*, indicating higher diffusivity of water through the specimens with respect to those submerged in moderate or alkaline mediums. Fourier transform infrared spectroscopy (FTIR) and scanning electron microscopy (SEM) analysis evidenced a weak interaction between the PLA matrix and the sisal fibers. Very limited degradation phenomena occur in our conditions: Despite some changes in the microstructure, the PLA backbone seems to be largely resistant to hydrolysis, almost regardless of the pH value and even at the highest sisal content.

## 1. Introduction

Poly(lactic acid) or polylactide (PLA) is one of the leading candidates to replace petrochemical-based plastics [1]. PLA is probably the most used biodegradable polymer in medical applications [2] or packaging [3] due to its thermoplastic processing capacity and its biodegradability under specific conditions. PLA, however, still has some challenges for commercial packaging applications due to its limited mechanical and barrier properties. Its performance has been improved significantly by tailoring its properties, adding specific compounds to accomplish with the established standards.

Natural fibers are widely used as reinforcement of PLA to improve its mechanical and thermal performance while maintaining its biodegradable nature. Several works deal with the selection of the fiber, its geometry, its optimum percentage, and disposition in the bio-polymeric matrix or its compatibility, among others [4]. Several fibers as flax [5,6] or kenaf [7,8] have been combined with PLA to achieve an optimized performance for specific user applications. Agave Sisalana (also known as sisal) with concentrations up to 30% *w*/*w* has been proved to be an adequate filler for PLA, improving its mechanical, thermal, and water uptake performance [9,10]. In addition, the characteristic properties of these bio-composites are retained after several recycling cycles, which would foster valorization techniques [11].

A remarkable advantage of these biopolymers is their biodegradability in various environments and for which their lifetime can be controlled. Aging of PLA in aqueous solutions at room temperature was previously investigated through immersion in distilled and seawater. Tests using distilled water [12,13,14] showed a decay on the mechanical properties of samples after one month and defined that the water absorption occurred according to Fick’s law. Aging in seawater was also evaluated [15,16,17,18] for which no significant changes were found in samples immersed up to 10 weeks. Temperature was shown to be a major parameter to accelerate the degradation [19], especially when approaching to glass transition. In addition, [9] studied the hydrothermal aging of the bio-composites PLA/sisal (with sisal up to 30% *w*/*w*) where a reduction of the molecular weight and an increase in the crystallinity of the samples was found for all samples.

Information regarding the behavior of PLA/sisal bio-composites when immersed in aqueous solutions at different pHs lacks in literature. This information becomes relevant and necessary if PLA bio-composites are to be applied to packaging elements containing acidic (i.e., pH = 2; lemon juice, vinegar), mildly acidic (i.e., pH = 6; milk), or mildly alkaline (i.e., pH = 8; baking soda, eggs) products. For this reason, the main objective of this work is to evaluate the influence of different pH in aqueous solutions on the water uptake, and on the chemical and morphological properties of pure PLA and PLA/sisal bio-composites. To our knowledge, the effect of treatment in solutions at different pHs in mild treatment conditions has not been reported for these materials, and its study can be useful from both an applicative and a fundamental point of view. We also attempt to reach a connection between morphological characterization at the macroscale level with spectroscopic characterization of these novel composites, whose properties have not been fully exploited yet. For this purpose, different characterization methods were applied. Scanning electron microscopy was used to evaluate changes in the morphology of the PLA and PLA-sisal composite materials after treatment in potential degradation conditions. Spectroscopies, namely infrared spectroscopy, were applied to characterize the polymer structure and to investigate interactions at the molecular level among the biodegradable polymer matrix and additives and compatibilizers having different chemical nature [20,21,22,23]. Finally, Fourier transform infrared spectroscopy was also used to evidence of possible chemical degradation at polymer surfaces after thermal or thermomechanical treatments [24,25]. 

## 2. Materials and Methods 

### 2.1. Materials and Sample Preparation

Poly(lactid acid) (PLA) 3251D was purchased from Natureworks (Minnetonka, MN, USA). The sisal fiber was supplied by Thai Royal project (Songkhla, Thailand). The processing of the initial bio-composites was detailed in a previous work [9]. Shortly, the initial samples were processed to obtain bio-composites with up to 30% in weight of sisal fibers and were labelled as PLA/PLA10/PLA20/PLA30, where the number indicates the weight percentage of sisal in the formulation. The degradation medium is indicated with a D (distilled water), S (seawater), PH2, PH6, and PH8 (pHs) in the samples labeling.

All the materials were mixed in an internal mixer (Brabender PL-2000 Plasti-Corder, Duisburg, Germany) for 5 min at 180 °C and 50 rpm of speed and subsequently pressed by compression moulding (PressesLabPro 400 Press, Fontijne Grotnes. Barendrecht, The Netherlands) by preheating to 200 °C for 2 min. Then, by applying a compression force of 150 kN during 2 min, under vacuum, 0.5 mm thick plates were obtained and used as specimens. 

### 2.2. Degradation Procedure

The bio-composite sheets were cut to specimen dimensions of 20 mm × 20 mm × 0.5 mm with thickness (*l*) 0.5 mm. The samples were then submerged (in triplicate) into distilled water, natural seawater (from Ligurian sea, sample at pH = 8), and aqueous solutions at different pHs (pH = 2, 6, and 8) at room temperature. Harsh degradation conditions, such as highly basic pH and/or heating at the glass point temperature were avoided in this study in order to possibly approach more common application conditions. The aqueous solutions at different pHs were prepared based on the formulation of buffer solutions according to [26]. 

After fixed periods of time, samples were removed from the solutions, weighed and submerged back into the corresponding solutions. The average content of absorbed water was calculated by triplicate by weight difference. A total of 60 samples were tested. 

An initial characterization of properties was performed to establish the baseline conditions of the bio-composites. Water uptake measurements were done every day until stabilization. FTIR and SEM analysis were carried out at the end of the testing period (i.e., 30 days). 

### 2.3. Analytic Monitoring of Degradation

#### 2.3.1. Water Uptake

The initial weight ($w_{0}$) was measured and recorded. Samples (in triplicate) were immersed in the aqueous mediums at room temperature. After certain periods of time, the specimens were removed, surface moisture gently wiped, and then weighed by means of an analytical Sartorius balance ($w_{t}$) with a precision of 0.1 mg. Then, the samples were submerged back to the aqueous medium. The absorbed water ($M_{t}$) was calculated according to (1):

(1) $$M_{t}=\;\frac{w_{t}-w_{0}}{w_{0}}\times 100$$

The saturation mass $M_{s}$ was calculated as $M_{t}$ at the equilibrium, taken as the value of the mass uptake at the asymptotic line. 

The diffusivity, sorption, and permeation were studied using the Fickian model. When considered plane sheet geometry, given $M_{t}$ at time t and $M_{s}$, Fick’s law can be simplified using Stefan’s approximation (2) [27]. This approximation is used for the initial stages of water uptake (i.e., $M_{t}$/$M_{s}$ < 0.5):

(2) $$\frac{M_{t}}{M_{s}}=\frac{4}{l}{\left(\frac{Dt}{\pi }\right)}^{1/2}$$

where D is the diffusion coefficient, calculated according to (3) from the plot $M_{t}$/$M_{s}$ as a function of the square root of time being θ the slope of the plot:

(3) $$D=0.0065\pi l^{2}\theta^{2}$$

The sorption coefficient (S) was calculated by (4):

(4) $$S=\;\frac{w_{s}}{w_{0}}$$

with $w_{s}$ the mass of absorbed water at equilibrium. 

The permeation coefficient (P) was calculated by (5):

(5) P=D×S

#### 2.3.2. Spectroscopic Characterization

Fourier transform infrared spectra in the mid-IR region were recorded using a FTIR spectrometer Thermonicolet 380 (DTGS detector, OMNIC^TM^ software, v. 7.2) equipped with ATR accessory (diamond crystal, ThermoFisher Scientific, Waltham, MA, USA). Each spectrum results from 100 scans and background in air. For each sample, three consecutive measurements were collected at different points on the platelet surfaces. Intensity ratios were calculated from average integrated area under specific absorption bands or from average peak height, measured at the band maximum. Features considered were centered at about 1750 cm^−1^ (carbonyl band) and about 1455 cm^−1^ (CH band) [24].

Near infrared spectra of the PLA and the composite layers were recorded in a Jasco V570 instrument in transmission mode (Jasco software 1.39.00) [28]. Several spectra of the sisal containing composites were also recorded for comparison by the same instrument using a diffuse reflectance accessory (spectra not shown).

#### 2.3.3. Morphological Characterization

Morphological characterization of PLA after the degradation procedure in distilled water and natural sea water was obtained with scanning electron microscopy (SEM) by using a Hitachi S–2500 instrument (Hitachi, Chiyoda, Tokyo, Japan) in the secondary electron at 10 KV. The samples were observed at 100×, 500×, and 2000× after coating with a nanometric gold film of about 30 nm obtained with the Polaron sputter coater (Thermo VG Scientific, East Grinstead, UK).

## 3. Results and Discussion

### 3.1. Macroscopic Appearance 

**Figure 1:**
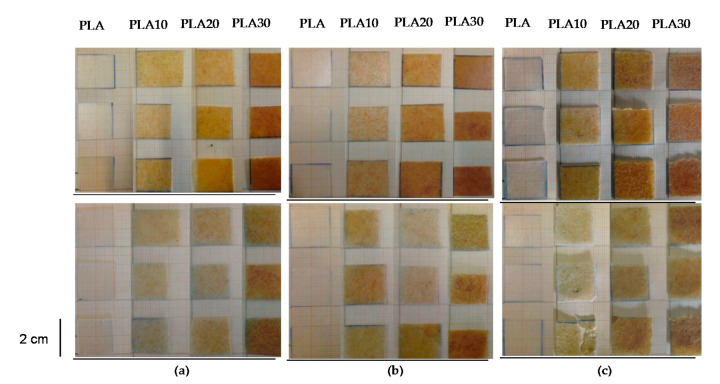
Macroscopic appearance of bio-composites at pH = 2 (**a**), pH = 6 (**b**), pH = 8 (**c**) after soaking for 0 days (above) and 30 days (below). Three specimens are reported for each composition.

The macroscopic appearance during the submersion time was assessed. Figure 1 shows the pictures of the samples as a function of the exposure time at the different aqueous mediums. In this series of samples, the influence of pH is considered. All bio-composites seemed to keep their physical consistency, probably due to the low testing temperature which did not promote significant structural changes as reported for high temperatures [9].

As expected, swelling phenomena were seen in most of the samples due to penetration of water into the bio-composite. These phenomena were more evident at increasing percentage of fibers because of the presence of a more hydrophilic component in the polymeric matrix and resulted independence from the pH.

PLA seems to be physically unaffected at the macroscale level by its immersion in any of the aqueous solutions regardless of the pH value. On the contrary, an evident opacification takes place when bio-composites are immersed in the different solutions, more evident at a higher pH. In general, the whitening of samples indicates that hydrolysis phenomena took place, thus confirming the existence of some degradation process [29]. This hypothesis is confirmed and its extension is deeply evaluated in the following FTIR discussion (Section 3.4.2).

### 3.2. Microscopic Characterization 

**Figure 2:**
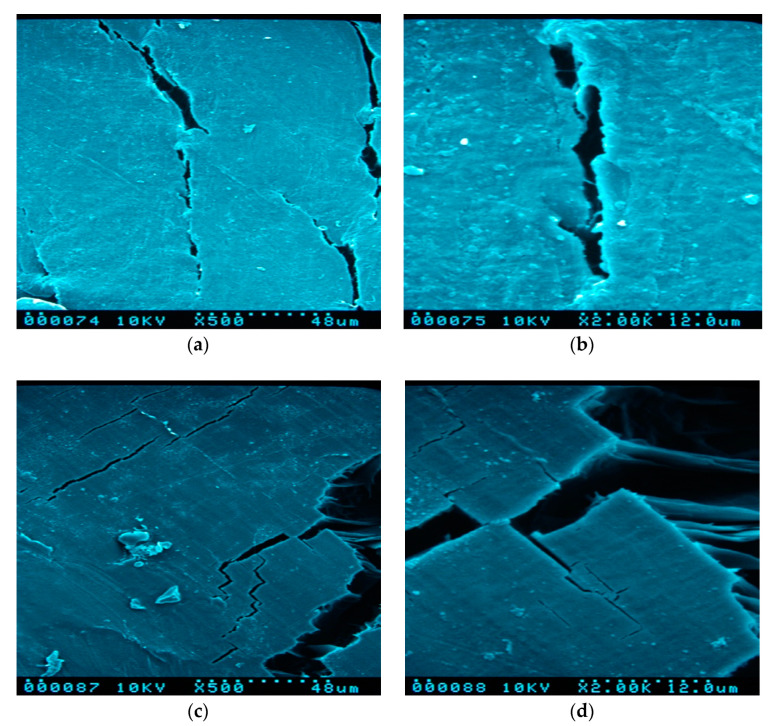

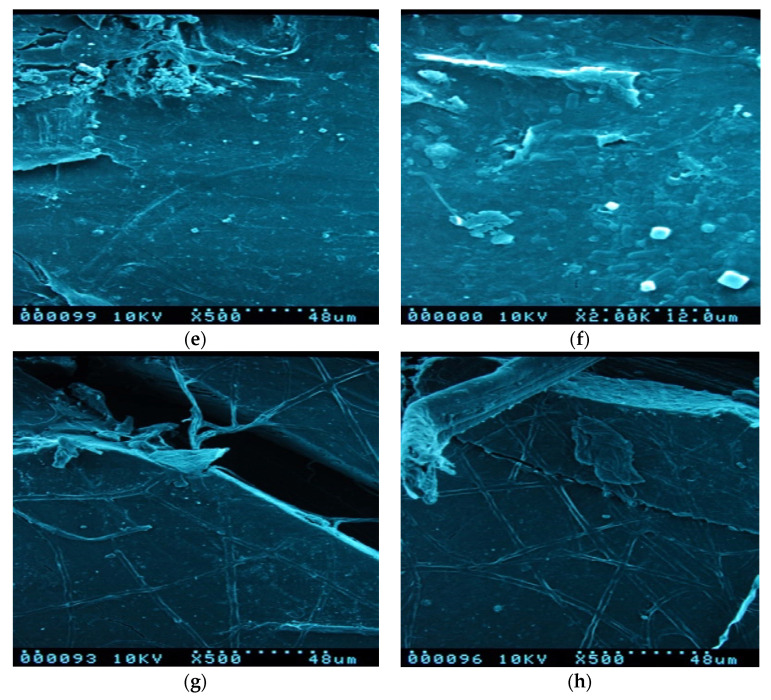
SEM images for PLA bio-composites: PLA before water soaking (**a**,**b**), PLA in distilled water (**c**,**d**), PLA in natural sea water (**e**,**f**), and PLA30 in distilled water (**g**,**h**). Panels (**a**,**c**,**e**,**g**) are referring at a magnification of 500×, panels (**b**,**d**,**f**,**h**) at a magnification of 2000×.

The microscopic changes due to degradation phenomena were assessed at the end of the exposure time. All the SEM images were taken on the surface of the samples at two different magnifications, 500× and 2000×, in order to have an overall vision of a portion of the specimen, concerning an area of about 100 um × 100 um, and more detailed evidence of the effect of the water at microscopic level. In some cases, macrocracks with sharp edges are present on the surface, as it is evident in Figure 2c,d,g,h, where deep crevices of about 10–20 um in width are coupled with thin cracks lower of 1 um, that can depart from the main rift or remain isolated. In other cases, the cracks are smaller and with a rounded border as in Figure 2a,b. If the presence of the cracks on the surface is a consequence of the internal stresses in a polymeric material at low toughness, but it can be considered generally independent by the water treatment, the surface roughness and the borders edges are influenced by the water action. It is possible to observe in fact as the immersion in distilled water produces a smoother surface (Figure 2c,d) with respect to one of the reference samples before the water soaking (Figure 2a,b). This phenomenon, which is more evident in the PLA without sisal (see Supplementary Materials, Figure S1), is probably due to the partial dissolution of the external layer of the polymer. This effect is less evident in the samples after degradation in natural sea water (Figure 2e,f), in which a superficial roughness is still present with several crystals of a cubic shape that can be ascribed to the nucleation of the salts in the water. The corresponding FTIR spectra confirming the deposition of salts is discussed in Section 3.4.2. The dissolution of the external layers of the polymer is more evident in PLA with a high degree of sisal (PLA30D), in which a mat of randomly disposed straight fibers emerges from the bulk of the polymeric system (Figure 2g,h). Figures S1–S3 in Supplementary Materials report on the SEM images of all PLA and composites samples immersed in distilled water and natural seawater as well as with SEM images of samples before soaking for comparison.

### 3.3. Water Uptake

**Figure 3:**
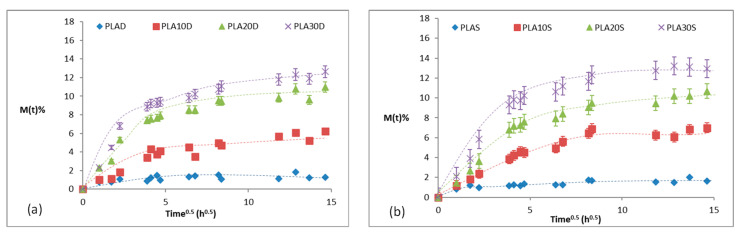

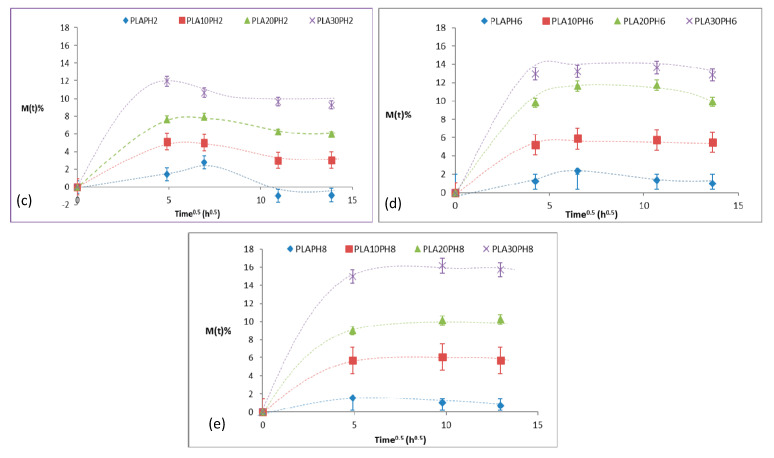
Water absorption of PLA and PLA-bio-composites submerged in: (**a**) distilled water, (**b**) seawater, (**c**) solution at pH = 2, (**d**) solution at pH = 6, and (**e**) solution at pH = 8.

Figure 3 presents the water absorption curves for PLA and its bio-composites submerged in distilled water, seawater, and in the different pH aqueous solutions.

**Figure 4:**
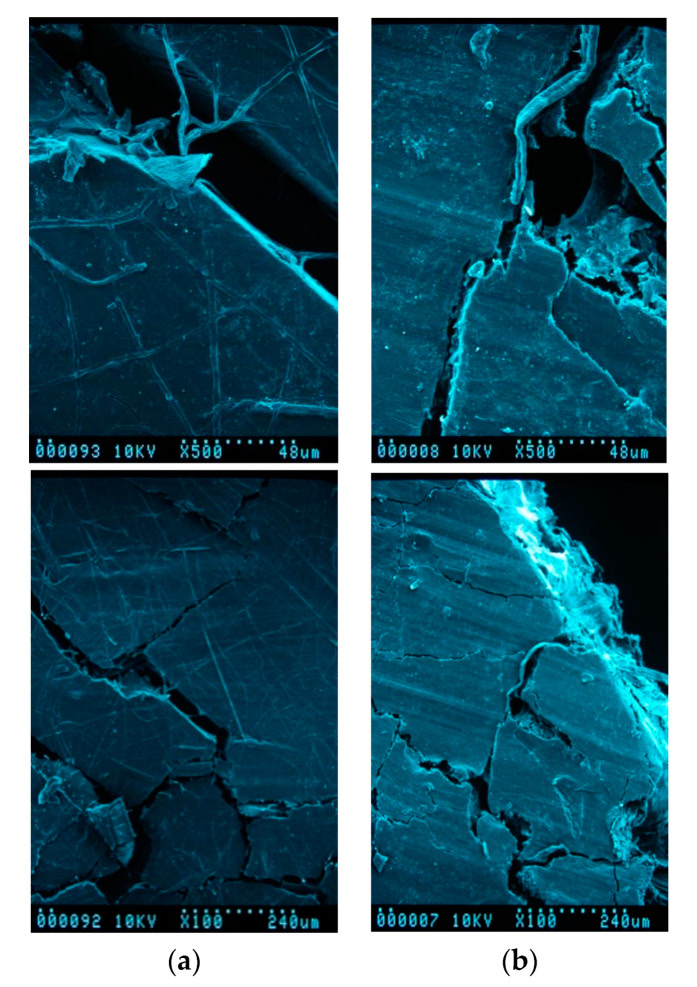
SEM images for bio-composites after submersion during 30 days in distilled water (**a**) and natural sea water (**b**).

In general, the bio-composites presented two well-differentiated stages for all mediums: (i) a rapid initial water uptake, with an increased rate with the increasing content of fibers; and (ii) an asymptotic saturation plateau. All samples showed a non-Fickian behavior after 10 days, not represented for lack of significance in the current calculations. The presence of surface microcracks through which water can flow in a preferential way and swell fibers was shown at larger degradation times, as it is possible to observe in Figure 4a (samples in distilled water after 30 days) and Figure 4b (samples in natural sea water after 30 days).

As expected, the water absorption increased with the increasing fiber content due to the affinity of hydroxyl groups of the cellulose in the sisal for water [30] as sisal easily bonds water molecules, resulting in higher water uptakes until reaching a maximum equilibrium value ($M_{s}$) which continues until *t*^1/2^ = 15 h in all samples.

The effect of pH was studied. The maximum water absorption was achieved at pH = 8 (~16%), indicating a more severe action of alkaline mediums on the samples. This behavior was also observed by Jung et al. [31] and Castro-Aguirre et al. [32] who showed that PLA is more easily degraded in strongly acidic/basic solutions and has a slow degradability at moderate pH conditions.

The water absorption at saturation ($M_{s}$), water diffusion coefficient (D), sorption coefficient (S), and permeation (P) coefficient for all bio-composites and degradation mediums are presented in Table 1.

The diffusivity of pristine PLA decreased with the addition of fibers. A sinusoid trend was observed with the increasing fiber content, being PLA20 the inflexion points for higher pHs. This behavior seems to contrast with what was expected: the hydrophilic nature of fibers should result in an increase in the diffusivity coefficient. Instead, the observed reduction of D could be indicative of changes in the internal microstructure, such as crystal growing phenomena [33] or hydrolytic chain scissions, as is discussed in Section 3.4.2. An increase in the fiber content could promote the formation of crystalline phases as sisal is known to act as a nucleation agent [9]. Fibers hinder the water advancement and decrease the transport paths for water molecules. Regarding the effect of pH, acidic mediums showed higher D, indicating higher diffusivity of water through the specimens with respect to those submerged in moderate or alkaline mediums. Overall, D was lower than that reported in literature for higher temperatures [9], mainly above the glass transition. 

The sorption coefficient, related to the equilibrium sorption of the aqueous medium, in general, increased with the increasing content of fibers (except for immersion in distilled water) and acidic mediums led to lower S with respect to moderate/alkaline solutions. 

Permeation, which describes the net effect of diffusion and sorption, in general, presented a decreasing trend with the increasing content of fibers, confirming the prevailing effect of diffusivity. In addition, the acidic pH showed the highest P values at all fiber contents, in accordance to the behavior shown for D.

### 3.4. Spectroscopic Characterization

#### 3.4.1. Non Degraded Bio-Composites

**Figure 5:**
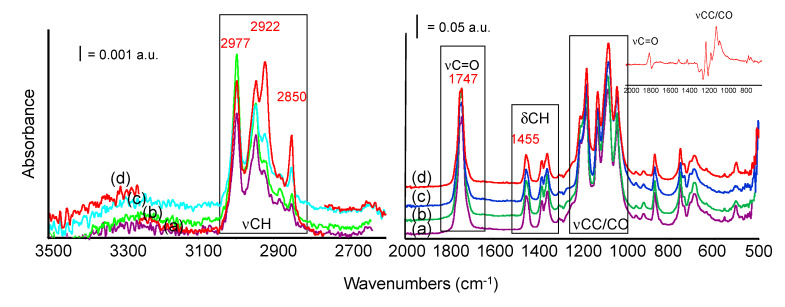
FTIR spectra of PLA and PLA bio-composites: pure PLA (a), PLA10 (b), PLA20 (c), PLA30 (d). Inset: subtraction spectrum: [spectrum PLA20]–[spectrum PLA].

The infrared spectra of pure PLA and PLA bio-composites with sisal are reported in Figure 5, ordered from bottom to top by increasing sisal content. The main bands detected are indeed those characterizing the PLA structure, for instance: the band centered at 1747 cm^−1^, corresponding to the C=O stretching mode of the ester group and the envelop of bands with maxima at 1180, 1081 cm^−1^ (C–O–C asymm and symmetric stretching modes), and 1042 cm^−1^ (CC stretching vibration mode). Another maximum at 1130 cm^−1^ is assigned to CH_3_ rocking mode. Weaker bands at 1455, 1380, and 1360 cm^−1^ are due to asymmetric and symmetric CH deformation modes in CH_3_ and CH groups, whose corresponding stretching modes are detected in the high frequency region of the spectrum at 2997, 2946, 2883, and 2850 cm^−1^ (weak). Few other very weak bands around 1300 cm^−1^ should also be assigned to CH deformation modes [34,35].

Two weak absorption bands evident at 1209 and 920 cm^−1^ and assigned to alkyl-carbonyl chain vibration and C–H bond vibration respectively, were related by Carrasco et al. [36] to the morphology of the PLA samples. Their disappearance should point out the decreased crystallinity of the sample. Changes in our composite formulation do not lead to any appreciable variation in this spectral region.

The bands described above are common to all the formulations analyzed in this work and the addition of sisal fibers only slightly affects the overall spectra, even at the highest sisal content (30% w). In the high frequency region of the spectra, some changes in the relative intensity of the bands occur, namely in the PLA20 and PLA30 samples spectra. For instance, if we evaluate the complex envelop of bands in the region 2950–2800 cm^−1^ (C–H stretching modes), the relative intensity of the component at 2922 cm^−1^ progressively increases at increasing sisal content, in comparison to the intensity of nearby bands evaluated in the same spectrum. The same effect can be detected, although less significant, for the component at 2850 cm^−1^ (Figure S4 Supporting Information). This means that, although these components can be already detected, weaker, in the spectrum of pure PLA, the addition of sisal changes relative intensities. For instance, similar features were assigned to symmetric and asymmetric methyl and methylene groups of side chains likely in the lignin structure of sisal [37,38] which, in our case, will overlap with features typical of PLA chains leading to an increased relative absorption in this region. Correspondingly, the relative intensity of the band at 2997 cm^−1^ (v_as_CH_3_) slightly decreases (although not proportionally) in samples PLA20 and PLA30, if compared to other bands in this region (i.e., bands at 2922 and 2850 cm^−1^). These observations could be explained by the decrease of the polylactic component, which exhibits the major contribution to the CH_3_ stretching bands in the composites spectra.

Moreover, a closer inspection of the subtraction spectrum reported in Figure 5, inset, also evidences some weak additional absorptions in the region 1560–1490 cm^−1^, possibly assigned to the ring stretching mode of the aromatic component in lignin and few positive absorptions between 1180 and 1030 cm^−1^ assigned to CO vibrational modes in cellulose, either to C–O stretching of secondary alcohols, or to C–O stretching of aliphatic ethers, strongly overlapped and masked by the absorptions of the PLA matrix [39]. A red shift of the C=O (ester) stretching band, appearing as a broad positive band centered around 1760 cm^−1^, could be explained by the interaction between the different fraction of the composites and the C=O group of polyester, likely through H-bonds.

**Figure 6:**
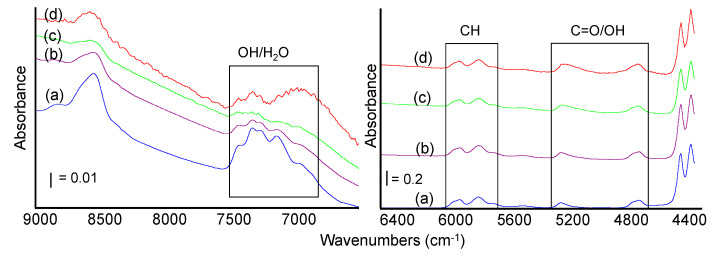
NIR spectra of PLA and PLA bio-composites: pure PLA (a), PLA10 (b), PLA20 (c), PLA30 (d).

In addition, spectra recorded in the near infrared region are very similar regardless of the different formulations (Figure 6). Although the discussion of NIR bands of PLA is complex, the assignation of the main bands in this region is proposed as follows, considering available literature data. Features in the region between 6000–5700 cm^−1^ are assignable to CH vibrational modes. In detail, bands of the first overtones of asymmetric and symmetric stretching vibrations of CH_3_ were reported at 5915 and 5785 cm^−1^, whereas the band at 5495 cm^−1^ is assigned to the first overtone of the CH stretching vibrations and the shoulder at 5680 cm^−1^ can be due to the combination band of stretching vibrations of the CH_3_ groups. The overall intensity of bands in the range 6000–5650 cm^−1^ as well as the splitting of the CH_2_ stretching band observed between 5980 and 5940 cm^−1^ were related to the ordered/disordered structure of the polymer [39].

The broad signal centered at 5270 cm^−1^ and tailing towards lower frequencies could correspond to the second overtones of the C=O stretching vibrations. The complex component at 4730 cm^−1^ could be assigned to combination modes of terminal OH groups [40].

Addition of sisal up to 30% wt leads to the formation of a shoulder at 4830 cm^−1^ and to an increase and broadening of the band at 5270 cm^−1^, (related to C=O vibrational modes but also to combination band of OH fundamental stretch and bending in cellulose and overtones of adsorbed water vibrational modes) [41,42]. At the highest sisal content, another broad absorption is growing just below 7000 cm^−1^ (first overtone of OH stretching mode in carbohydrates) and features in the range 7400–7100 cm^−1^ are significantly changed. These findings agree with an increased content of OH groups in the samples due to the cellulosic component in sisal and to the related increased uptake of water by H-bonds. The broadening of the band related to the C=O stretching could also be considered further evidence of the H-bonds between the PLA chain and the sisal fraction.

#### 3.4.2. Treated Samples

**Figure 7:**
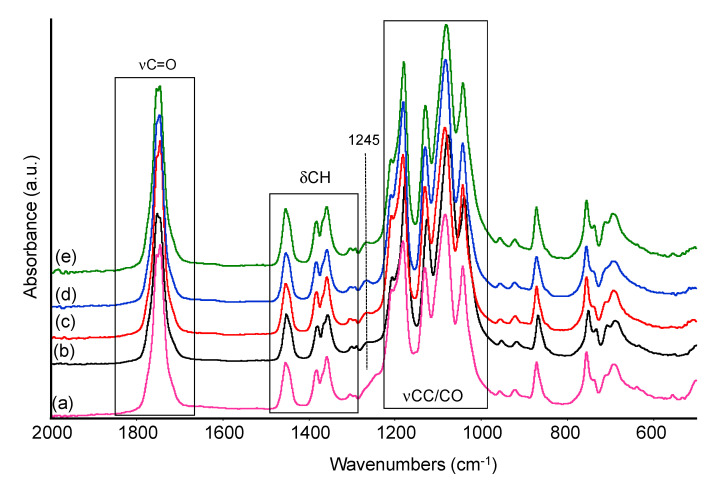
FTIR spectra of untreated PLA (a), PLA after 30 days treatment in distilled water (b), at pH 2 (c), at pH 6 (d), at pH 8 (e).

**Figure 8:**
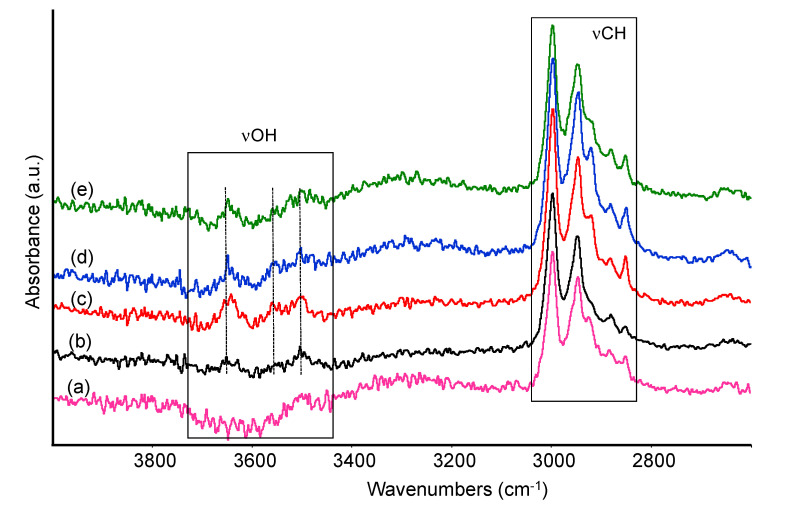
FTIR spectra of untreated PLA (a), PLA after 30 days treatment in distilled water (b), at pH 2 (c), at pH 6 (d), at pH 8 (e). High frequency region.

The same treated samples were analyzed by ATR spectroscopy in the mid-IR region after prolonged soaking in aqueous solutions, in order to evaluate possible differences in functional groups. Spectra of samples PLA and PLA20 are reported in Figure 7 and Figure 8 after treatment in distilled water and in water solutions at different pH at the end of the testing period (i.e., 30 days) as indicated in Section 2.2.

As for pure PLA sample, after treatment in water and at pH 2, 6, 8, the main polymer bands in the low frequency region seem to be unchanged. However, a closer inspection of the spectra recorded at different pH evidences that the component at 1245 cm^−1^, related to the C–O–C stretching mode of the ester, and a weak absorption at 1150 cm^−1^, likely another C–O stretching mode, almost disappear. Correspondingly, weak bands appear in the OH stretching region near 3600–3550 cm^−1^ and 3500 cm^−1^ (Figure 8). Similar features were assigned to O–H stretching modes of hydroxyl and carboxylic acid terminal groups of PLA and reported to increase in intensity following polymer chain scission [23]. These observations suggest, at least in samples treated at different pHs, some limited hydrolysis of the ester bond in the PLA chain, leading to the formation of few free alcoholic and carboxylic groups. The intensity of these bands, normalized with the intensity of the CH stretching modes, slightly increases at pH 2 and pH 8. In parallel, only small changes in the range 1760–1720 cm^−1^, typical of C=O stretching modes, were detected, for instance, a slight shift in wavenumbers and/or enlargement of the signal. The relative intensity of the carbonyl band is evaluated and discussed in the following section.

**Figure 9:**
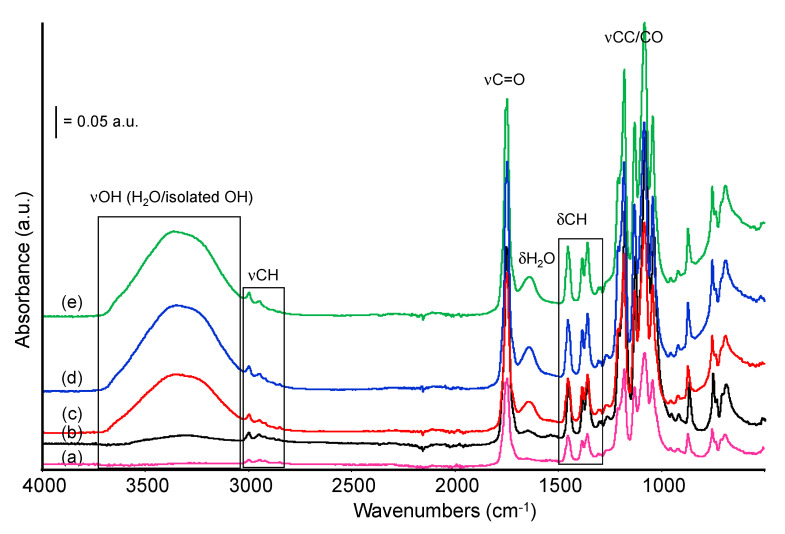
FTIR spectra of untreated PLA20 (a), PLA20 composite after 30 days treatment in distilled water (b), at pH 2 (c), at pH 6 (d), at pH 8 (e).

Adding sisal fibers to the formulation has, as a first macroscopic effect, the increase in the water uptake which leads to a noticeable increase of the spectroscopic bands diagnostic of water: OH stretching modes in the range 3500–3000 cm^−1^, H_2_O deformation modes at 1650 cm^−1^, and calibration mode appearing as an increased absorption below 1000 cm^−1^. The same effect is detected after soaking either in pure water either in water solution at different pH values, as exemplified for sample PLA20 (Figure 9). Roughly, the intensity of the IR bands due to adsorbed water is increasing at increasing sisal content, in agreement with the quantitative analysis of water uptake reported in the previous Section 3.3. The formation of hydrolysis products is likely also in the bio-composites, as suggested by the analysis of the spectra in Figure 9, which show additional small absorptions near 3640 cm^−1^, detected as shoulder of the main band of OH stretching mode. These features will be again assigned to free OH groups arising from ester hydrolysis, as reported for the pure PLA sample. NIR spectra of the same samples also evidenced for the composite sample a huge increase of bands due to overtones and combination modes of OH groups fundamentals, particularly in the range 5300–4600 cm^−1^, in agreement with data reported in the previous paragraph (spectra not reported). Moreover, in this spectral region, signal of free OH groups and COOH groups were reported to fall, strongly overlapped with OH groups from the cellulose and hemicellulose fraction and adsorbed water, thus the strong increase in intensity of these bands we observed in the NIR spectra can be also due to the presence of some hydrolysis products. 

Our results partially agree with literature findings on fiber-based composites. The presence of hydrophilic lignocellulosic fibers, has been reported to favor the hydration of the sample, which is considered the first step in the following breaking of the ester bond and, finally, in the chemical degradation or biodegradation processes of the polyester chain in soils [43,44,45]. However, in our composites, there is not clear evidence of an increased formation of degradation products detectable by IR analysis in comparison to pure PLA.

**Figure 10:**
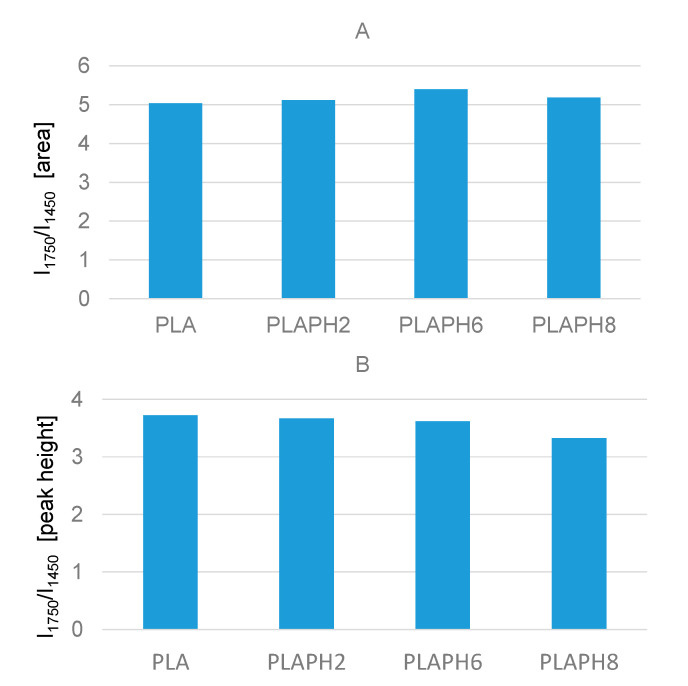
Pure PLA samples. Intensity ratio I_1750_/I_1455_ evaluated as integrated area (**A**) and as peak height (**B**).

In their work on PLA degradation at increasing temperatures, Cuadri et al. proposed the analysis of the relative intensities of the ester band, which is the intensity of the band at 1745 cm^−1^ (C=O stretching), normalized using the intensity of the band at 1450 cm^−1^ (CH_3_ deformation mode) as the internal standard [24]. Chain scission reactions, associated to thermo-oxidative and thermo-mechanical degradation, were reported to lead to an increase in this ratio, due to the formation of other carbonyl-containing groups in anhydrides and carboxyl compounds, whereas thermal degradation did not allow significant changes in the calculated intensity ratio. On the other side, degradation by hydrolysis should lead to a decrease in this ratio, explained by the random disappearance of the ester linkage in the chain and to the corresponding formation of carboxylic groups in oligomers and monomers [24]. Therefore, we decided to evaluate the same parameter to investigate possible changes in chemical structure of the polymer after long time soaking in aqueous solutions. In a first step, the ratio [I = intensity of carbonyl band (about 1750 cm^−1^)/intensity of CH band (about 1455 cm^−1^] was calculated for pure PLA samples, considering both integrated area and peak height (Figure 10).

On average, the relative intensity of the C=O band as the integrated area slightly increases following the soaking, and a trend of this effect corresponding to the different pH values cannot be defined. A likely explanation is that the minimal amount of hydrolysis suggested by the appearance of new OH groups should imply the formation of carboxylic acids whose C=O stretching modes fall in a very narrow frequency region, overlapped with the strong C=O band around 1750 cm^−1^, therefore affecting values of integrated intensity.

We cannot exclude the formation of new carbonyl-containing groups resulting from PLA degradation and functional groups rearrangements, although these phenomena should be limited at room temperature. Subtraction spectrum [spectrum PLA pH2]–[spectrum PLA] points out the growth of new components above 1745 cm^−1^ and negative bands around 1740 and 1250 cm^−1^, to support this hypothesis (Figure S5 Supporting Information).

On the other side, considering the same ratio referred to the height of the main ester peaks, treatments in water decreases this ratio, suggesting some hydrolysis of the group and this is especially evident at pH = 8 as it was also evident from the discussion in Section 3.3.

**Figure 11:**
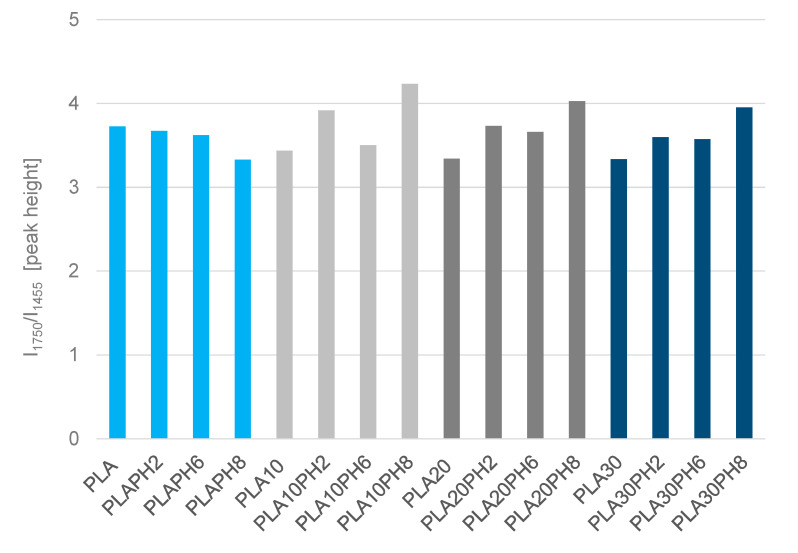
Band intensity ratio of pristine and treated samples I_1750_/I_1455_ (peak height).

The intensity ratio I_1750_/I_1455_ was evaluated also for sisal containing samples after different immersion in different degradation mediums, assuming that the addition of fibers does not significantly affect the bands examined for this study. Moreover, for the samples containing sisal fiber, the evaluation of the integrated intensity in this spectral region could be affected by the growing of nearby bands associated to adsorbed water, thus the intensity ratio was evaluated as height ratio (Figure 11).

Indeed, the presence of fibers showing similar IR bands extensively overlapped with PLA bands affects these evaluations (Figure S6 Supporting Information). Nevertheless, although a clear trend cannot be defined, it seems that there is an average increase in the relative intensity of the carbonyl band in all the bio-composite systems. Two explanations are possible: a limited formation of hydrolysis products whose diagnostic bands fall in a very similar frequency range as the untreated samples, and a morphological effect, i.e., the degradation of the surface and the consequent formation of some cracks induced by water which further exposes PLA and sisal fibers to the spectroscopic analysis. In this case, the surface should present heterogeneous regions where the ratio PLA/sisal changes, thus also altering the intensity ratio of IR bands. SEM results further support the latter hypothesis (see Section 2.2).

Possibly, the most significant changes are detected considering the composite samples after treatment at pH 8. Interestingly, this is the pH at which the maximum water adsorption was reported (Table 1) as well as a noticeable opacification (see Section 2.1).

As for peaks in the low frequency spectral region, soaking in aqueous solutions at different pHs, the sisal composites lead to a small increase of the relative intensities of peaks in the region 1100–1000 cm^−1^, especially in PLA20 and PLA30 samples, regardless of the specific pH value. This effect can be due to the presence of CO and CC stretching modes of functional groups belonging to the carbohydrate fraction of the fiber. This effect is in line with the proposed morphological effect, i.e., the degradation of the surface and the consequent formation of some cracks induced by water [29], which further exposes PLA surface and sisal fibers.

Pure PLA and composite samples were analyzed also after treatment at room temperature in natural sea water (Figure S7 Supporting Information), in agreement with experiments reported in the literature [18]. Beside the well-known effect of increased hydration of the sample revealed by a broad IR band in the high frequency region, consistent with the discussion reported above, some changes can be detected in the spectra, namely, few broad absorptions appear below 1750 cm^−1^, overlapped to bands due to the polymer matrix. These features are mainly related to deposition of salts, such as carbonate species or oxide/hydroxide species, in agreement with microscopy data reported in Section 3.2. Unfortunately, the presence of these additional absorptions in the mid IR region hinders the evaluation of band intensity as it was proposed in the previous discussion section.

In sum, the spectroscopic data discussed above, suggest that the addition of fibers to the pristine PLA polymer matrix produced small but significant changes in the main IR bands in shape or position in the spectra of composite materials. It was possible to detect a small increase in the intensity of CH stretching modes in the MID IR region, likely arising from the lignin component of the fiber, and, correspondingly, an increase of bands due to OH groups, from the cellulose and hemicellulose fraction as also evidenced by NIR analysis. The slight shift of bands related to carbonyl and ether groups suggested a weak interaction occurring between the two moieties of the bio-composites without deeply affecting the chemical structure of the PLA matrix. These results are in agreement with the SEM micrograph that evidenced a heterogeneous surface structure with well-defined fibers in the polymer matrix, clearly detected at the highest sisal contents. There is no spectroscopic evidence of changes in PLA crystallinity resulting in the composites from sisal addition.

The addition of sisal, corresponding to the increased hydrophilic fiber content, significantly increased water absorption in the samples, as clearly shown in the results of water uptake. This effect is qualitatively highlighted by ATR spectra in the region 3500–3200 cm^−1^ and 1630 cm^−1^ and by near infrared analysis and it has been reported for both pure water and mildly acidic and basic solutions.

Soaking pure PLA samples in aqueous solution at room temperature resulted in a very limited hydrolysis of PLA ester bonds to terminal alcohols and carboxylic acids, as suggested by the appearance of new OH groups and the complexity of the C=O band in the FTIR spectra. Apparently, treatments at pH 2 and pH 8 are those more significantly affecting PLA structure. This small effect, leading to some changes in the polymer microstructure can partially explain the observed reduction of the diffusion coefficient *D*.

Limited hydrolysis phenomena could be reported also for sisal composites, whose ATR spectra show some new OH features after treatment in aqueous solutions strongly overlapped with the IR absorption of the adsorbed water molecules. The quantification of hydrolysis products by spectroscopic analysis following literature reports is not straightforward, due to the possible formation of carbonyl-containing byproducts, whose IR absorptions are superimposed. Moreover, the morphological changes detected by SEM after soaking of samples in distilled water, that is dissolution of external polymer layers and emerging of fibers, will also contribute to change the surface exposed to IR analysis. Considering these limitations, at room temperature, degradation processes appear quite limited and, in spite of some changes in the microstructure, the PLA backbone seems to be largely resistant to hydrolysis, even at the highest sisal content corresponding to the highest water uptake in our experimental conditions.

## 4. Conclusions

PLA and sisal-PLA bio-composites were prepared and studied by spectroscopic and microscopic techniques, as such and following immersion at room temperature in different degradation mediums and monitored during a period of 30 days. Morphological and chemical evaluations allow us to propose the following conclusions.

- In the composites, weak interaction occurs between the two moieties, without deeply affecting the structure of the PLA matrix;
- Water absorption increased with the increasing hydrophilic fiber content, regardless of the immersion medium, whereas the diffusivity, *D*, of PLA decreased with the addition of fibers, possibly explained by the emerging of sisal fibers during soaking. On the other side, acidic mediums showed higher *D*, indicating higher diffusivity of water through the specimens in these conditions with respect to those submerged in moderate or alkaline mediums;
- Finally, FTIR spectroscopy and SEM analysis evidenced that very limited degradation phenomena occur in our conditions: the PLA backbone seems to be resistant to hydrolysis almost regardless of the pH value and even at the highest sisal content.

## Figures and Tables

**Table 1:** Water absorption at saturation ($M_{s}$), water diffusion coefficient (D), sorption coefficient (S), and permeation coefficient (P) for all bio-composites and degradation mediums.

Sample	$M_{s}\;(\%\;\mathrm{wt})$	D (cm^2^/s) × 10^8^	S (g/g)	P (cm^2^/s) × 10^8^
	**Distilled Water**			
PLA	1.1	-	-	-
PLA10	5.8	0.4	0.6	0.4
PLA20	10.4	0.1	2.0	0.2
PLA30	12.5	0.1	1.1	0.1
	**Natural Seawater**			
PLA	1.8	2.2	0.2	2.3
PLA10	7.0	0.7	0.7	0.8
PLA20	10.3	0.7	1.0	0.7
PLA30	13.0	0.7	1.3	0.8
	**pH = 2**			
PLA	−1.0	-	-	-
PLA10	3.0	1.8	0.3	1.9
PLA20	6.1	1.3	0.6	1.4
PLA30	9.5	1.3	0.9	1.4
	**pH = 6**			
PLA	1.2	1.2	0.1	1.2
PLA10	5.7	0.9	0.6	1.0
PLA20	11.7	0.8	1.2	1.0
PLA30	13.5	1.0	1.3	1.2
	**pH = 8**			
PLA	0.7	2.2	0.1	2.2
PLA10	6.1	0.7	0.6	0.8
PLA20	1.1	0.6	1.0	0.7
PLA30	16.3	0.7	1.6	0.8

## Supplementary Materials

The following are available online at https://www.mdpi.com/1996-1944/13/18/3974/s1, Figure S1: SEM images for PLA bio-composites before water soaking, at 100× (row 1), 500× (row 2) and 2000× (row 3) magnifications, Figure S2: SEM images for PLA bio-composites after water soaking in distilled water, at 100× (row 1), 500× (row 2) and 2000× (row 3) magnifications, Figure S3: SEM images for PLA bio-composites after water soaking in natural see water, at 100× (row 1), 500× (row 2) and 2000× (row 3) magnifications, Figure S4: FTIR spectra of PLA bio-composites: PLA10 (a), PLA20 (b), PLA30 (c). CH stretching region, Figure S5: FTIR subtraction spectrum [spectrum PLAPH 2]–[spectrum PLA], Figure S6: FTIR spectra of PLA and PLA composites after treatment in aqueous solution (pH = 2): pure PLA (a), PLA10 (b), PLA20 (c), PLA30 (d), Figure S7: FTIR spectra of PLA and PLA composites after treatment in natural seawater: pure PLA (a), PLA10 (b), PLA20 (c), PLA30 (d).
