# Supplementary material for: Influence of the Degradation Medium on Water Uptake, Morphology, and Chemical Structure of Poly(Lactic Acid)-Sisal Bio-Composites
Source: Materials (Basel). 2020 Sep 8;13(18):3974. doi: 10.3390/ma13183974 (PMC7557377; doi:10.3390/ma13183974)
Supplement: Supplementary file 1 [file materials-13-03974-s001.pdf]

materials

MDPI

Supplementary Information

Influence of the Degradation Medium on Water Uptake, Morphology, and Chemical Structure of Poly(Lactic Acid)-Sisal Bio-Composites

Cristina Moliner *, Elisabetta Finocchio *, Elisabetta Arato, Gianguido Ramis and Alberto Lagazzo

Department of Civil, Chemical and Environmental Engineering (DICCA), University of Genoa, via all'Opera Pia 15, I-16145, Genova (Italy); elisabetta.arato@unige.it (E.A.); gianguidoramis@unige.it (G.R.); alberto.lagazzo@unige.it (A.L.)

* Correspondence: cristina.moliner@edu.unige.it (C.M.); Elisabetta.Finocchio@unige.it (E.F.), Tel.: +39-010 3352919

Received: 31 July 2020; Accepted: 04 September 2020; Published: date

PLA (initial)	PLA10 (initial)	PLA20 (initial)	PLA30 (initial)

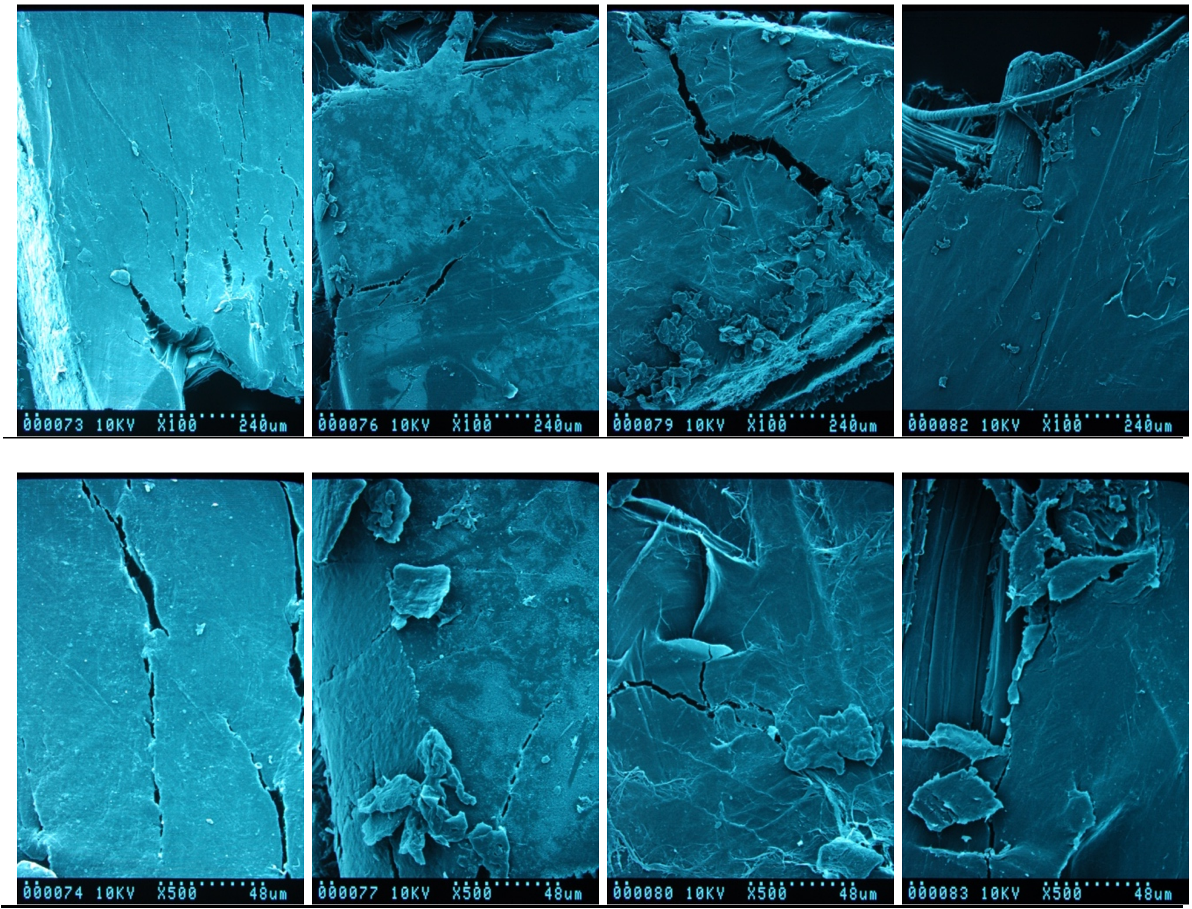

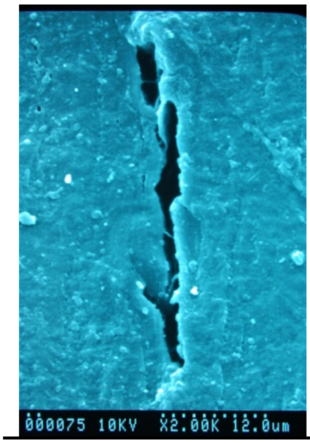	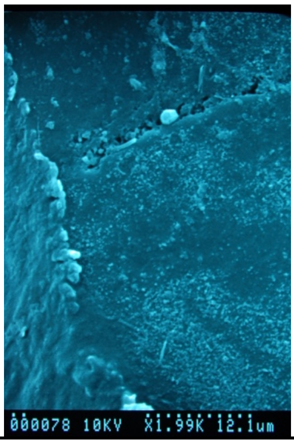	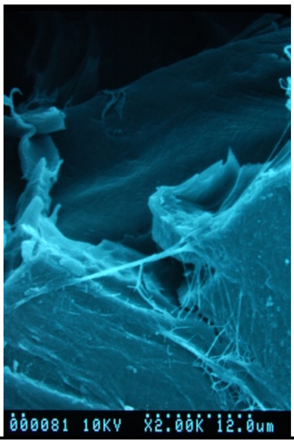	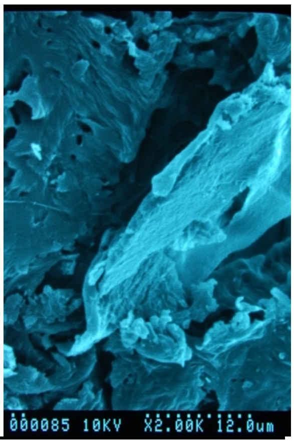

Figure S1. SEM images for PLA bio-composites before water soaking, at 100× (row 1), 500× (row 2) and 2000× (row 3) magnifications.

PLA (distilled water)	PLA10 (distilled water)	PLA20 (distilled water)	PLA30 (distilled water)
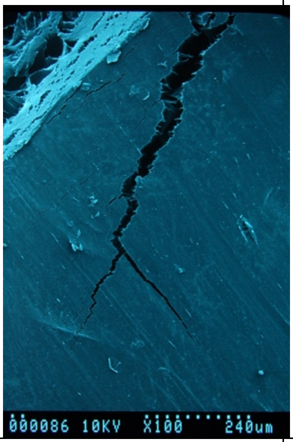	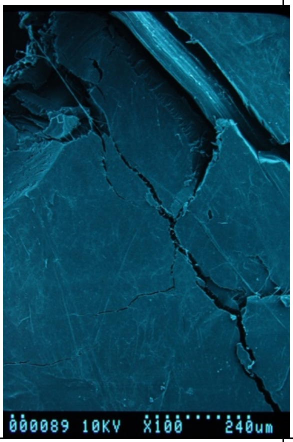	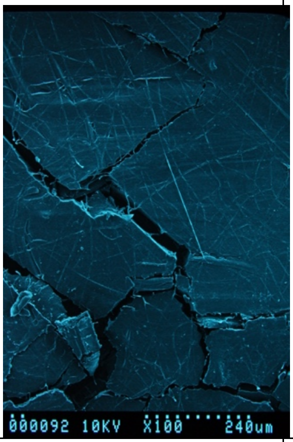	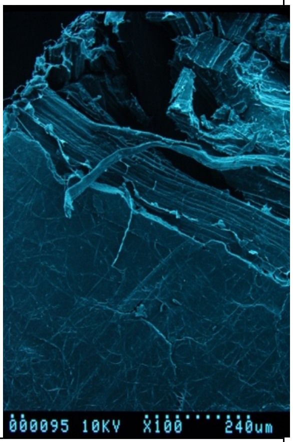
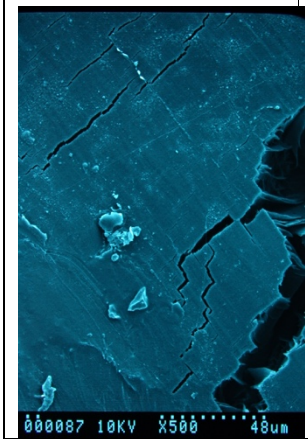	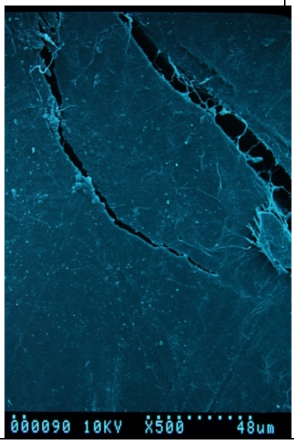	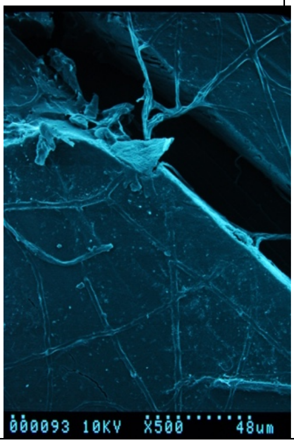	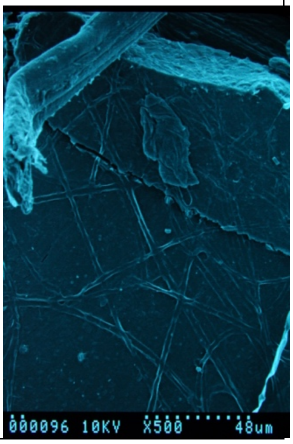

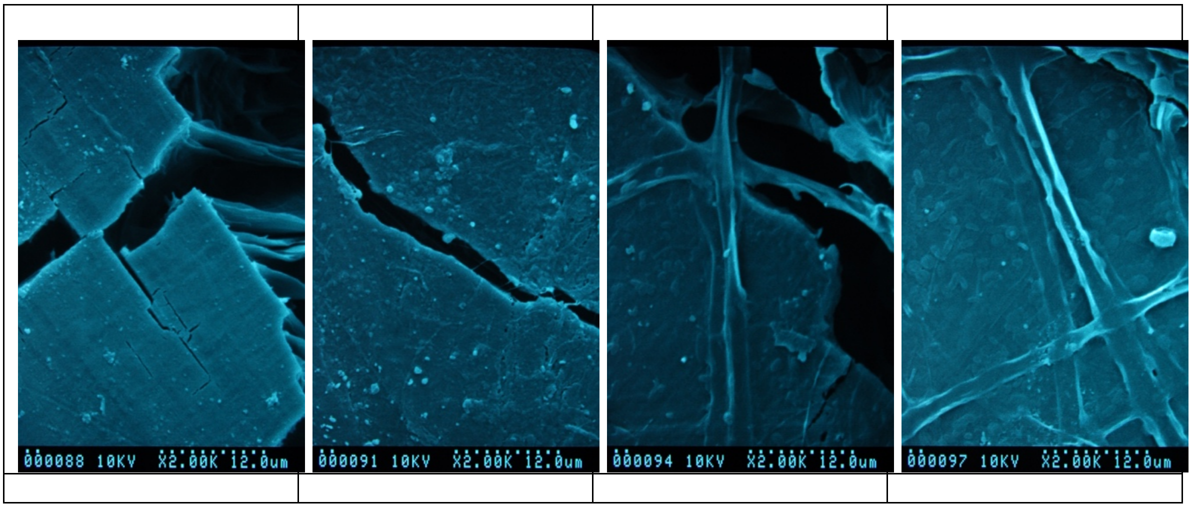

Figure S2. SEM images for PLA bio-composites after water soaking in distilled water, at 100× (row 1), 500× (row 2) and 2000× (row 3) magnifications.

PLA (sea water)	PLA10 (sea water)	PLA20 (sea water)	PLA30 (sea water)

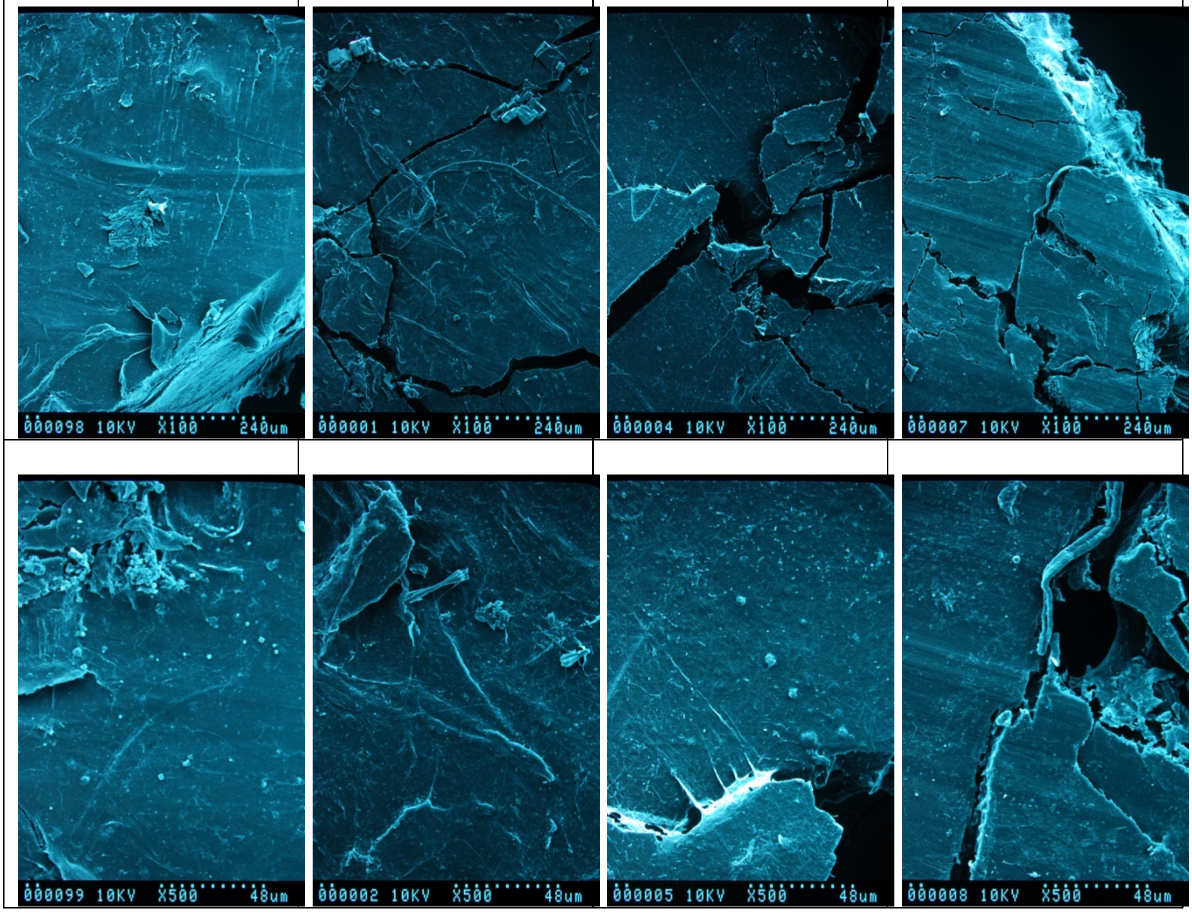

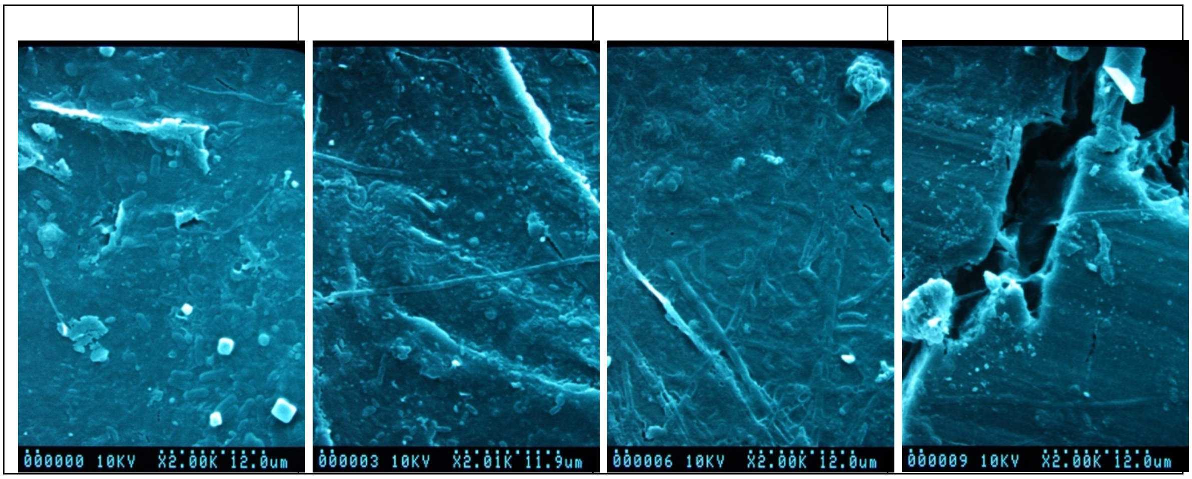

Figure S3. SEM images for PLA bio-composites after water soaking in natural see water, at 100× (row 1), 500× (row 2) and 2000× (row 3) magnifications.

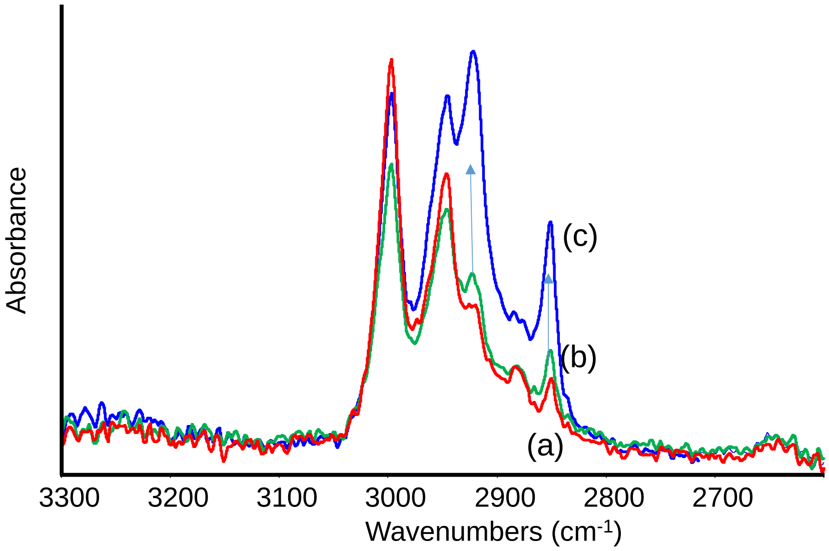

Figure S4. FTIR spectra of PLA bio-composites: PLA10 (a), PLA20 (b), PLA30 (c). CH stretching region.

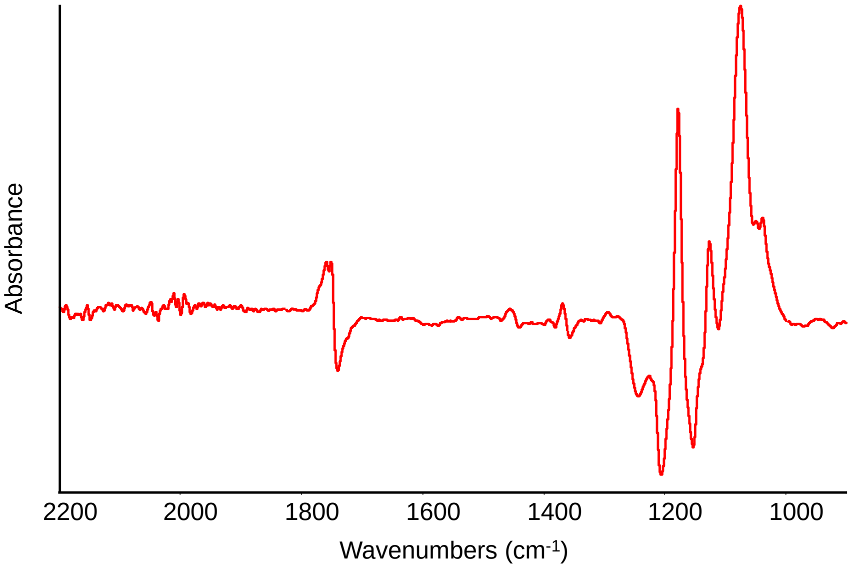

Figure S5. FTIR subtraction spectrum [spectrum PLA pH2]–[spectrum PLA].

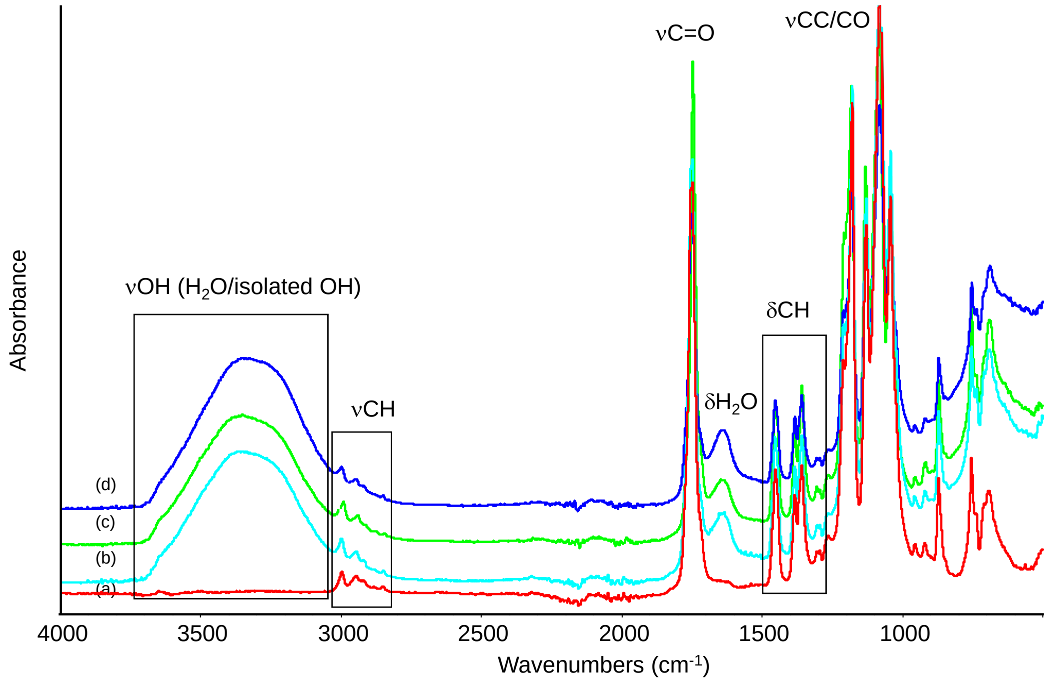

Figure S6. FTIR spectra of PLA and PLA composites after treatment in aqueous solution (pH = 2): pure PLA (a), PLA10 (b), PLA20 (c), PLA30 (d).

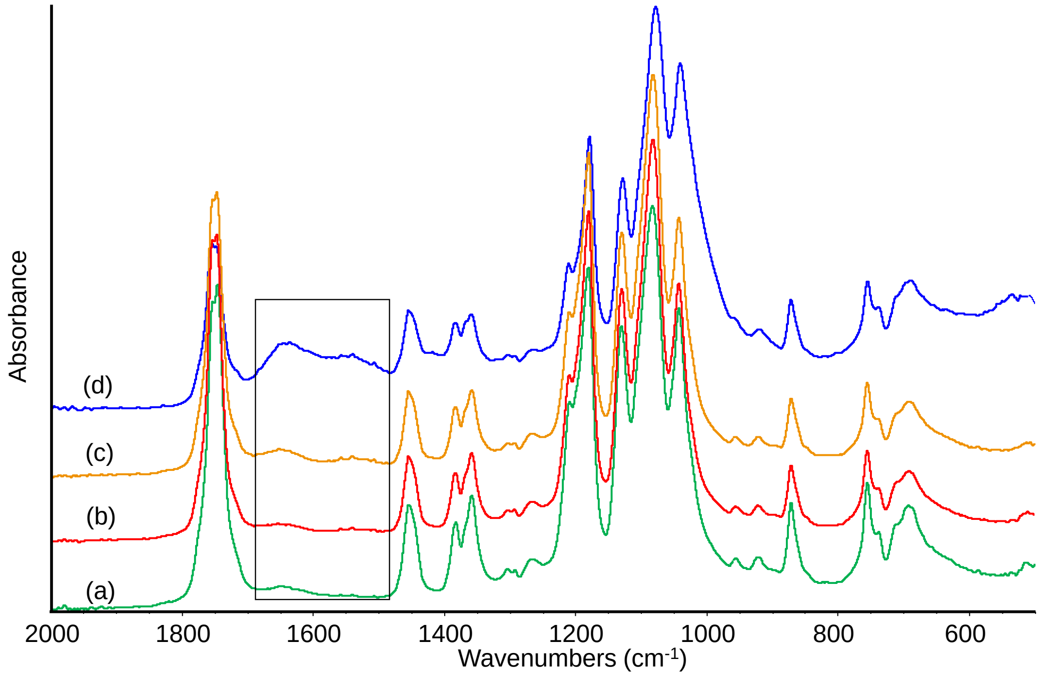

Figure S7. FT IR spectra of PLA and PLA composites after treatment in natural seawater: pure PLA (a), PLA10 (b), PLA20 (c), PLA30 (d).

© 2020 by the authors. Submitted for possible open access publication under the terms and conditions of the Creative Commons Attribution (CC BY) license (http://creativecommons.org/licenses/by/4.0/).
